# Nonsurgical Orthodontic Intervention of a Severe Class II Case Accompanied by Posterior Crossbite Using a Miniscrew-Assisted Straight Wire Technique

**DOI:** 10.1155/2019/5696370

**Published:** 2019-10-03

**Authors:** Abdulkarim A. Hatrom, Ahmed R. Afify, Ali H. Hassan

**Affiliations:** ^1^Ministry of Health, Makkah, Saudi Arabia. P.O. Box 4137, Makkah 24359, Saudi Arabia; ^2^Orthodontic Department, Faculty of Dentistry, King Abdulaziz University, P.O. Box 80209, Jeddah 21589, Saudi Arabia; ^3^Orthodontic Department, Faculty of Dentistry, Mansoura University, Mansoura, Egypt; ^4^Alfarabi Private College for Dentistry and Nursing, P.O. Box 23643, Jeddah 21589, Saudi Arabia

## Abstract

Class II malocclusion in nongrowing patients is a great challenge in treatment especially if the degree of malocclusion is severe. In such cases, the use of miniscrews for orthodontic camouflage can produce results comparable to that of orthognathic surgery. This case report presents an adult patient with severe Class II malocclusion combined with bilateral posterior crossbite, deep bite, a crowded lower arch, and a history of extraction of the lower right first molar. The treatment involved upper arch expansion by a quad helix appliance followed by the extraction of the right and left upper 1^st^ premolars. A miniscrew-assisted straight wire technique was used to close the extraction space and reduce the overjet. Lower molar protraction was done to close the previous extraction space in the lower arch. At the end of treatment, overjet was reduced, lower arch crowding was relieved, lip competency was established, and the wide buccal corridor was reduced with a pleasing smile and normal facial proportions.

## 1. Introduction

Patients suffering from Class II malocclusion are usually affected by their problems which make them the most common cases that seek orthodontic treatment [1]. Angle Class II Division 1 is usually characterized by maxillary protrusion and/or mandibular retrusion. This is usually accompanied by protrusion of the upper anterior teeth, narrow upper arch, and incompetent upper lip [1, 2]. The presence of such problems in adult patients is challenging because these patients usually have high expectations regarding the results of treatment [3].

In nongrowing patients, this malocclusion can only be treated by one of two methods; orthodontic camouflage which usually involves the extraction of two upper premolars [4] or even upper and lower premolars or orthognathic surgery to reposition the mandible and/or the maxilla in a normal position. It was shown that patient satisfaction and perception of outcome with camouflage treatment are nearly the same to those achieved by orthognathic surgery and that orthodontic camouflage showed fewer functional and temporomandibular joint problems [5].

With the recent advancement in anchorage devices and the introduction of miniscrews in orthodontics, orthognathic-like results can be achieved by utilizing such devices [6]. In patients with Class II malocclusion, it was shown that a maxillary incisors retraction of 8.2-9.3 mm can be achieved by using miniscrews [7, 8].

The following case had severe Class II malocclusion with bilateral posterior crossbite, deep bite, a crowded lower arch, and a history of extraction of the lower right first molar which was managed by orthodontic camouflage instead of orthognathic surgery.

## 2. Diagnosis and Treatment Planning

**Figure 1:**
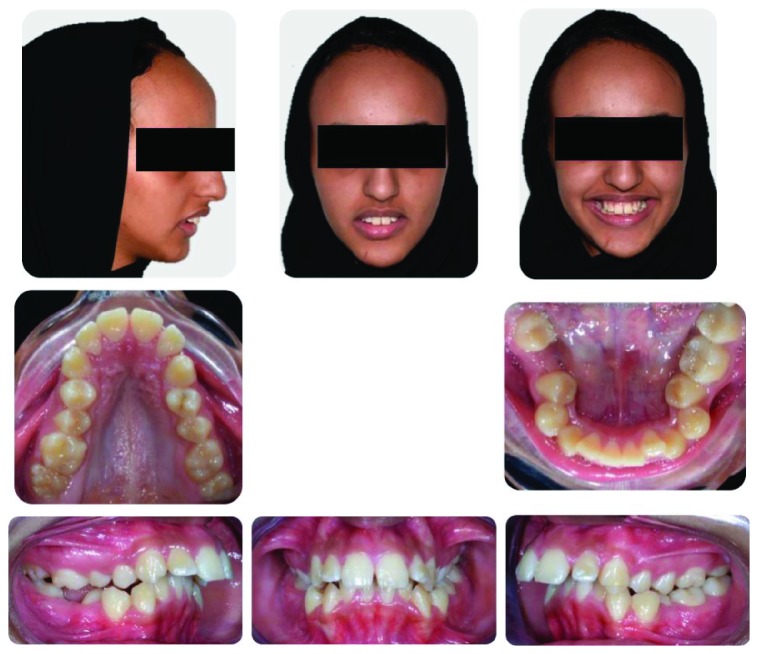
Pretreatment extraoral and intraoral photographs of the patient.

**Figure 2:**
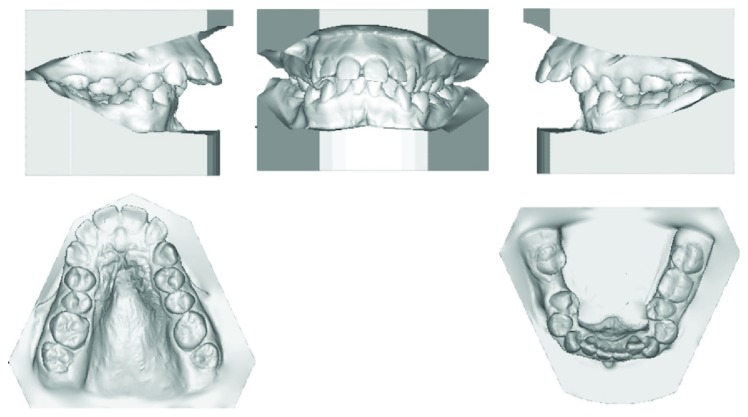
Pretreatment models.

A female patient aged 18 years presented to the Orthodontic Department of King Abdulaziz University with a chief complaint of sticking out front teeth. She had no significant medical history while her dental history showed extraction of the lower right first molar 1 year ago. Clinical extraoral examination showed a dolichocephalic facial type, convex profile with proclined upper incisors, and incompetent lips. Intraoral examination revealed Class II malocclusion with bilateral posterior crossbite, deep bite, and increased overjet. Both the upper and lower arches were narrow while the lower arch showed a moderate amount of crowding. Smile analysis showed that she had a very wide buccal corridor due to narrow arches and slight gingival display which is acceptable at this age (Figures 1 and 2).

**Figure 3:**
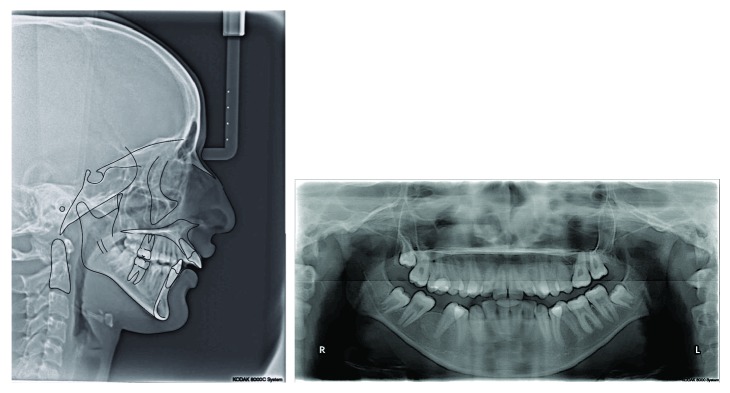
Pretreatment cephalometric and panoramic radiograph.

Cephalometric analysis revealed a Class II skeletal relationship (ANB angle = 6.4°) and a steep mandibular plane angle (36.2°). Regarding dental measurement, it was found that she had proclined upper incisors (U1-PP = 116.6°) and retroclined lower incisors (L1-MP = 82°) (Figure 3).

A panoramic radiograph revealed a missing lower right first molar, an upper left first molar, and no sign of root resorption. The upper and lower third molars were still in the stages of root formation. No caries or periapical lesion was seen (Figure 3).

### 2.1. Treatment Objectives

In order to obtain a well-balanced facial esthetics, adequate smile parameters and good stable occlusion of the following treatment objectives were planned: (1) correcting the proclination of the upper incisors which in turn will reestablish the correct position of the relatively short and incompetent upper lip, (2) widening the upper arch to close the wide buccal corridor and enhance the smile esthetics, and (3) closing the extraction space of the lower right first molar to establish a stable occlusion.

### 2.2. Treatment Plan

First, it is necessary to expand the upper arch preferably by utilizing quad helix. Then, extraction of the upper right and left first premolars after completion of arch expansion and alignment of teeth was done. Protraction of lower right second molar was done to close the space of the lower right first molar and allow the eruption of the third molar in its place. And finally, upper removable wraparound retainers and lower fixed retainer were used to retain the established results.

### 2.3. Treatment Progress

The treatment was initiated with a banding and bonding procedure using modified bidimensional preadjusted edgewise brackets, 0.018-inch slots in the incisors and canines, and 0.022-inch slots in the premolars and molars (3M Unitek, Monrovia, CA, USA). Roth prescription was combined with cementation of the quad helix appliance. Leveling and alignment were achieved with a straight wire technique which was used in the following sequence: 0.012^″^ Niti, 0.014^″^ Niti, 0.016^″^ Niti, and 0.016 × 0.025^″^ Niti followed by 0.018 × 0.022^″^ SS wire.

**Figure 4:**
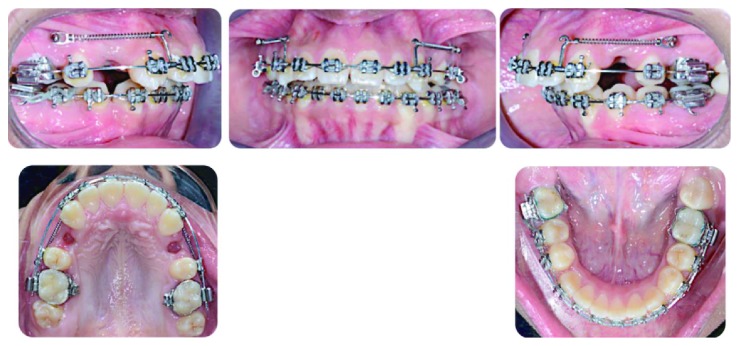
Progress photographs.

After finishing arch expansion in the upper arch, extraction of the upper right and left 1^st^ premolars was done. Under local anesthesia, insertion of two miniscrews 1.6 mm in diameter and 8 mm in length (3 M Unitek, Monrovia, CA, USA) between the upper 2^nd^ premolar and the first molar on both sides was done. An en-masse anterior tooth retraction by using a nickel-titanium closed coil spring with a force value of 250 gram force was performed on each side, and the nickel-titanium closed coil spring was attached to a power arm 5 mm in length positioned mesial to the canine. A rigid stainless steel wire (0.018 × 0.022^″^) was inserted during retraction. The force was repeated every 3 weeks until the overjet was reduced, and extraction spaces were completely closed. Finishing was done utilizing intermaxillary elastics until satisfactory functional and static occlusion were achieved. Active treatment time was 28 months, and the patient was instructed to wear the acrylic retainer in the upper arch, and a fixed bonded retainer was used in the lower arch (Figure 4).

### 2.4. Treatment Results

**Figure 5:**
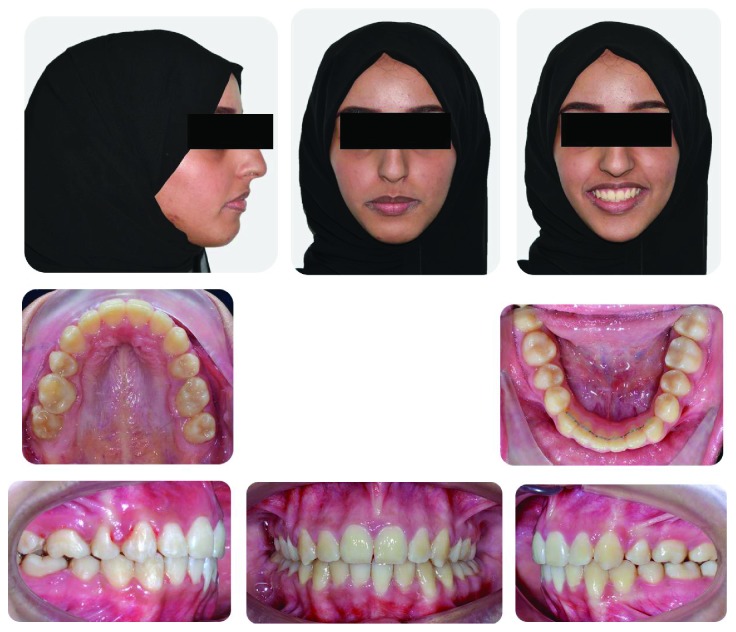
Posttreatment photographs of the patient.

**Figure 6:**
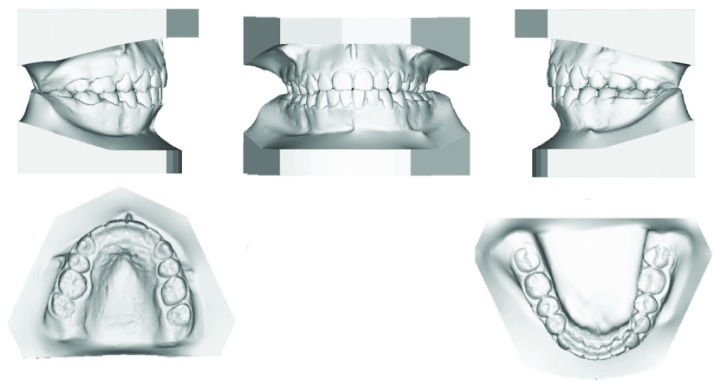
Posttreatment models.

**Figure 7:**
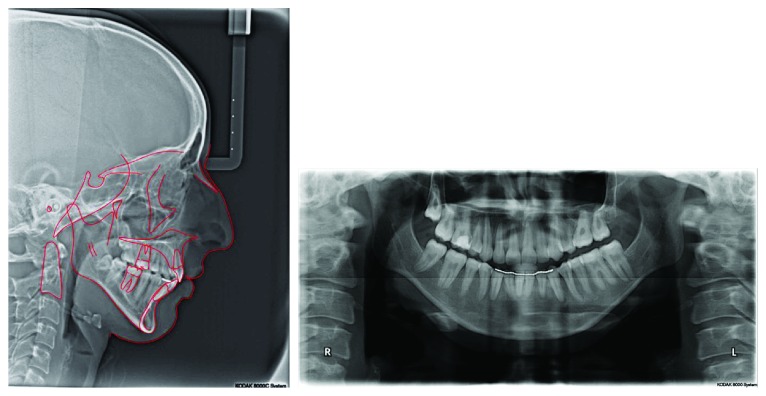
Posttreatment cephalometric and panoramic radiograph.

**Figure 8:**
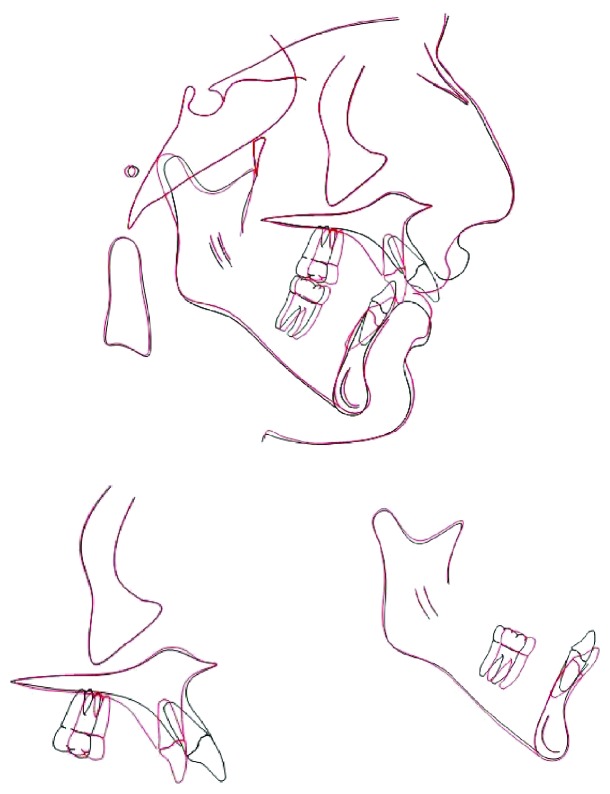
Posttreatment cephalometric superimposition.

Amazing enhancement in both facial and smile esthetics was established. Lip competency was reached, and the buccal corridor was reduced with a pleasing smile and normal facial proportions (Figures 5 and 6). Regarding cephalometric analysis, mainly dental changes were observed after the end of treatment. Upper incisors became more retroclined (U1-PP = 93°) while the lower incisors became more proclined (L1-MP = 95°) making all the dental measurements within the normal range. The surrounding bone and periodontal tissues were found to be healthy during and after the treatment without any evidence pocket formation or bone loss. The teeth remained caries free with no signs of root resorption when viewed on the panoramic radiograph (Figures 7 and 8).

## 3. Discussion

Adult patients with severe Class II malocclusion may be treated by camouflage or a combination of orthodontic and orthognathic surgeries depending on the severity of malocclusion [9]. The main goal of treatment by orthodontic camouflage is to mask the marked skeletal discrepancy by dental compensations. In Class II malocclusion when extractions are needed, they are usually done in the maxillary first premolars to correct the proclination of the upper incisors [5]. This is usually followed by en-masse retraction of the upper incisor with absolute or maximum anchorage to close the extraction space and reduce overjet. This will lead to flattening of the nasolabial angle improvement lip position [10].

However, it was suggested that orthognathic surgery has better esthetic outcomes which are observed in the marked improvement of the soft tissue profile and high stability or the results obtained [11]. On the other hand, other studies proved that the amount of incisor retractions which are depending on the type of anchorage used is the determining factor for the resultant changes in lip position and soft tissue profile [4, 12]. This opinion was also supported with a study by Mihalik et al. that showed that orthodontic camouflage by extraction of the upper premolars produced stable results comparable to that of orthognathic surgery [5].

Orthognathic surgery is barely accepted by patients for many reasons such as high cost, risk of infection, fear of general anesthesia, and the risk of this invasive procedure [13, 14]. This patient refused orthognathic surgery due to all of the abovementioned reasons, and orthodontic camouflage was the possible line of treatment.

In this case, the expansion of the upper arch was done utilizing the quad helix appliance to gain more space and to close the wide buccal corridor seen during smiling. The choice of the quad helix appliance was due to its effect in decreasing the mandibular plane angle in hyperdivergent patients [15].

The extraction of the upper right and left first premolars was done to allow for the retraction of the proclined upper incisors and to reduce the overjet with concomitant restoration of lip competency. This was done by using miniscrews with en-masse anterior tooth retraction utilizing nickel-titanium closed coil springs on each side with a force value of 150 gram force as recommended by Upadhyay et al. [16]. The power chain was attached to a power arm 5 mm in length positioned mesial to the canine to apply the force directly at the center of resistance and to avoid tipping [17]. A rigid stainless steel wire (0.018 × 0.022^″^) was utilized to control the torque of incisors during retraction.

The protraction of the lower right second molar was done to close the space of the lower right first molar and allow the eruption of the third molar in its place. Molar protraction has the advantage of preservation of the tooth structure when compared with a fixed bridge and reduced cost when compared with dental implants. Also, this method reduces the risk of impaction of the third molar.

## References

[1] Carvalho Ferreira F. P., Barbosa Lima A. P., de Paula E. . C. M., Ferreira Conti A. C. . C., Valarelli D. P., de Almeida-Pedrin R. R. (2016). Orthodontic protocol using mini-implant for class II treatment in patient with special needs. *Case Reports in Dentistry*.

[2] Shioya S., Arai K. (2017). Dentoskeletal morphology of adult class II division 1 and 2 severe deep overbite malocclusions. *Orthodontic Waves*.

[3] van Wezel N. A., Bos A., Prahl C. (2015). Expectations of treatment and satisfaction with dentofacial appearance in patients applying for orthodontic treatment. *American Journal of Orthodontics and Dentofacial Orthopedics*.

[4] Qamruddin I., Shahid F., Alam M. K., Zehra Jamal W. (2014). Camouflage of severe skeletal class II gummy smile patient treated nonsurgically with mini implants. *Case Reports in Dentistry*.

[5] Mihalik C. A., Proffit W. R., Phillips C. (2003). Long-term follow-up of class II adults treated with orthodontic camouflage: a comparison with orthognathic surgery outcomes. *American Journal of Orthodontics and Dentofacial Orthopedics*.

[6] Raposo R., Peleteiro B., Paço M., Pinho T. (2018). Orthodontic camouflage versus orthodontic-orthognathic surgical treatment in class II malocclusion: a systematic review and meta-analysis. *International Journal of Oral and Maxillofacial Surgery*.

[7] Kuroda S., Yamada K., Deguchi T., Kyung H. M., Takano-Yamamoto T. (2009). Class II malocclusion treated with miniscrew anchorage: comparison with traditional orthodontic mechanics outcomes. *American Journal of Orthodontics and Dentofacial Orthopedics*.

[8] Kim K., Choi S. H., Choi E. H., Choi Y. J., Hwang C. J., Cha J. Y. (2017). Unpredictability of soft tissue changes after camouflage treatment of class II division 1 malocclusion with maximum anterior retraction using miniscrews. *The Angle Orthodontist*.

[9] Proffit W. R., Phillips C., Douvartzidis N. (1992). A comparison of outcomes of orthodontic and surgical-orthodontic treatment of class II malocclusion in adults. *American Journal of Orthodontics and Dentofacial Orthopedics*.

[10] Upadhyay M., Yadav S., Nanda R. (2010). Vertical-dimension control during en-masse retraction with mini-implant anchorage. *American Journal of Orthodontics and Dentofacial Orthopedics*.

[11] Tucker M. R. (1995). Orthognathic surgery versus orthodontic camouflage in the treatment of mandibular deficiency. *Journal of Oral and Maxillofacial Surgery*.

[12] Scott Conley R., Jernigan C. (2006). Soft tissue changes after upper premolar extraction in class II camouflage therapy. *The Angle Orthodontist*.

[13] Cunningham S., Hunt N., Feinmann C. (1995). Psychological aspects of orthognathic surgery: a review of the literature. *The International Journal of Adult Orthodontics and Orthognathic Surgery*.

[14] Rivera S. M., Hatch J. P., Dolce C., Bays R. A., van Sickels J. E., Rugh J. D. (2000). Patients’ own reasons and patient-perceived recommendations for orthognathic surgery. *American Journal of Orthodontics and Dentofacial Orthopedics*.

[15] Endo S., Yamada W., Shundo I., Kobayashi Y., Komatsuzakit A., Endo T. (2016). Short-term treatment effects of the quad-helix appliance on dentofacial morphology of hyperdivergent patients. *Australian Orthodontic Journal*.

[16] Upadhyay M., Yadav S., Patil S. (2008). Mini-implant anchorage for en-masse retraction of maxillary anterior teeth: a clinical cephalometric study. *American Journal of Orthodontics and Dentofacial Orthopedics*.

[17] Tominaga J.-y., Tanaka M., Koga Y., Gonzales C., Kobayashi M., Yoshida N. (2009). Optimal loading conditions for controlled movement of anterior teeth in sliding mechanics: a 3D finite element study. *The Angle Orthodontist*.

